# The rise and fall of fasciculations in amyotrophic lateral sclerosis

**DOI:** 10.1093/braincomms/fcaa018

**Published:** 2020-02-20

**Authors:** James A Bashford, Aidan Wickham, Raquel Iniesta, Emmanuel M Drakakis, Martyn G Boutelle, Kerry R Mills, Chris E Shaw

**Affiliations:** f1 UK Dementia Research Institute, Department of Basic and Clinical Neuroscience, Maurice Wohl Clinical Neuroscience Institute, Institute of Psychiatry, Psychology and Neuroscience, King’s College London, London, UK; f2 Department of Bioengineering, Imperial College London, London, UK; f3 Department of Biostatistics and Health Informatics, King’s College London, London, UK

**Keywords:** amyotrophic lateral sclerosis, fasciculation, EMG, biomarker, nerve excitability

## Abstract

Amyotrophic lateral sclerosis is a devastating neurodegenerative disease with a median survival of 3 years from symptom onset. Accessible and reliable biomarkers of motor neuron decline are urgently needed to quicken the pace of drug discovery. Fasciculations represent an early pathophysiological hallmark of amyotrophic lateral sclerosis and can be reliably detected by high-density surface electromyography. We set out to quantify fasciculation potentials prospectively over 14 months, seeking comparisons with established markers of disease progression. Twenty patients with amyotrophic lateral sclerosis and five patients with benign fasciculation syndrome underwent up to seven assessments each. At each assessment, we performed the amyotrophic lateral sclerosis-functional rating scale, sum power score, slow vital capacity, 30-min high-density surface electromyography recordings from biceps and gastrocnemius and the motor unit number index. We employed the Surface Potential Quantification Engine, which is an automated analytical tool to detect and characterize fasciculations. Linear mixed-effect models were employed to account for the pseudoreplication of serial measurements. The amyotrophic lateral sclerosis-functional rating scale declined by 0.65 points per month (*P* < 0.0001), 35% slower than average. A total of 526 recordings were analysed. Compared with benign fasciculation syndrome, biceps fasciculation frequency in amyotrophic lateral sclerosis was 10 times greater in strong muscles and 40 times greater in weak muscles. This was coupled with a decline in fasciculation frequency among weak muscles of –7.6/min per month (*P* = 0.003), demonstrating the rise and fall of fasciculation frequency in biceps muscles. Gastrocnemius behaved differently, whereby strong muscles in amyotrophic lateral sclerosis had fasciculation frequencies five times greater than patients with benign fasciculation syndrome while weak muscles were increased by only 1.5 times. Gastrocnemius demonstrated a significant decline in fasciculation frequency in strong muscles (−2.4/min per month, *P* < 0.0001), which levelled off in weak muscles. Fasciculation amplitude, an easily quantifiable surrogate of the reinnervation process, was highest in the biceps muscles that transitioned from strong to weak during the study. Pooled analysis of >900 000 fasciculations revealed inter-fasciculation intervals <100 ms in the biceps of patients with amyotrophic lateral sclerosis, particularly in strong muscles, consistent with the occurrence of doublets. We hereby present the most comprehensive longitudinal quantification of fasciculation parameters in amyotrophic lateral sclerosis, proposing a unifying model of the interactions between motor unit loss, muscle power and fasciculation frequency. The latter showed promise as a disease biomarker with linear rates of decline in strong gastrocnemius and weak biceps muscles, reflecting the motor unit loss that drives clinical progression.

## Teaser

Employing high-density surface electromyography alongside a novel analytical algorithm, this longitudinal study explores the interactions between fasciculation firing, neuronal loss and disease progression in amyotrophic lateral sclerosis. Showing most promise as a potential biomarker, fasciculation frequency is postulated to rise steadily and then fall rapidly over the course of disease.

## Graphical Abstract

**Graphical Abstract:**
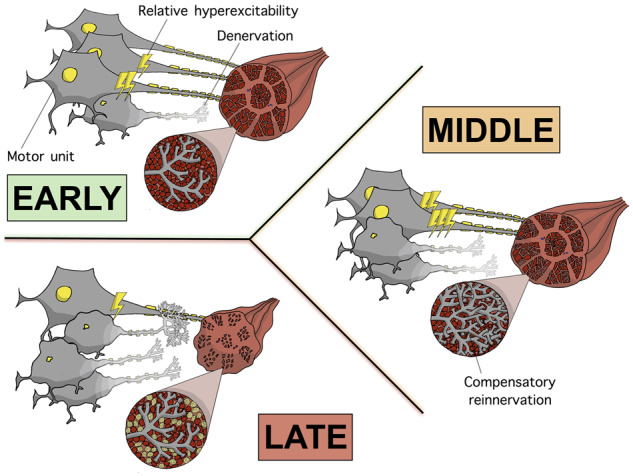

## Introduction

Amyotrophic lateral sclerosis is a devastating neurodegenerative disease with a median survival of just 3 years from symptom onset (Alonso *et al.*, 2009; Al-Chalabi and Hardiman, 2013). Accessible and reliable biomarkers that are sensitive to motor neuron decline are urgently required to quicken the pace of drug discovery (Benatar *et al.*, 2016). Drug trials could become shorter and less expensive if well-calibrated biomarkers, demonstrating target engagement with the putative therapy, could increase the homogeneity of the enrolled disease cohort. Biomarkers that directly measure the pathophysiology of amyotrophic lateral sclerosis, including motor unit (MU) number estimates (Van Dijk *et al.*, 2010; Shefner *et al.*; 2011; Neuwirth *et al.*, 2015; Neuwirth *et al.*, 2017), transcranial magnetic stimulation (Mills and Nithi, 1997; Vucic *et al.*, 2013b; Geevasinga *et al.*, 2019) and excitability testing (Burke *et al.*, 2001; Kanai *et al.*, 2006), have shown great promise. However, none of these techniques has yet been introduced as a mainstream clinical biomarker. A common disadvantage among these methods is the need for reasonably complex acquisition protocols that require significant neurophysiological and analytical expertise, thereby limiting their widespread translation into outcome measures in clinical trials (Vucic and Rutkove, 2018). We have been driven by this observation and recognize the advantages of a tool that maximizes simplicity and automation.

Fasciculations are a pathophysiological hallmark of amyotrophic lateral sclerosis (Mills, 2010; De Carvalho *et al.*, 2017). Most relevant of all, their presence precedes the onset of muscle weakness (De Carvalho, 2000), warranting diagnostic emphasis in the Awaji consensus criteria (De Carvalho *et al.*, 2008; Costa *et al.*, 2012). High-density surface electromyography (HDSEMG) records fasciculations in a non-invasive way (Drost *et al.*, 2007), generating data sets with high temporal and spatial resolution, prime for systematic quantification (Kleine *et al.*, 2008; Sleutjes *et al.*, 2016). We have recently developed Surface Potential Quantification Engine (SPiQE), which is an automated analytical tool designed to detect and characterize fasciculation potentials from resting HDSEMG (Bashford *et al.*, 2019). Validation demonstrated an overall classification accuracy of 88% at identifying fasciculations from raw HDSEMG, while additional refinements of the method identified and excluded unrelaxed muscle activity (Bashford *et al.*, 2020). SPiQE is capable of analysing 30-min recordings, producing simple outputs related to fasciculation frequency (FF), amplitude, inter-fasciculation intervals (IFIs) and data quality.

To our knowledge, only one study has specifically assessed FF in patients with amyotrophic lateral sclerosis longitudinally using surface EMG (De Carvalho and Swash, 2016). The authors did not detect a change in FF in the first dorsal interosseous over 12 months, despite a significant decrease in the neurophysiological index, which correlates with clinical (Cheah *et al.*, 2011) and subclinical disease progression (Escorcio-Bezerra *et al.*, 2018). We wanted to address this question again in the light of an updated methodological approach, significantly expanding the data set to include two muscle types (with bilateral data collection) and longer recordings of 30-min duration. We anticipated that the noise-responsive model underpinning SPiQE would mitigate variations in data quality, making comparisons between recordings more reliable. Compared with previous static approaches to fasciculation identification, which rely on the same amplitude inclusion threshold across every recording (De Carvalho and Swash, 2013), the new dynamic model would allow for an improved fasciculation detection rate, particularly when data quality was good.

A MU comprises the motor neuron cell body, axon, terminal branches and connecting muscle fibres (Burke, 1967). It is well established from needle EMG studies that amyotrophic lateral sclerosis leads to a process called chronic partial denervation (De Carvalho and Swash, 2013). This means that as MUs succumb to the disease and die, surviving MUs are instructed to sprout and branch to reinnervate orphaned muscles fibres. This is an evolutionary, compensatory mechanism designed to maintain muscle power in the face of a reduced MU pool. In amyotrophic lateral sclerosis, a reinnervating MU steadily acquires new muscle fibres and consequently produces MU action potentials of larger amplitude, longer duration and greater complexity (Krarup, 2011; Bokuda *et al.*, 2016). However, due to the relentless loss of MUs in amyotrophic lateral sclerosis, this process of reinnervation cannot maintain muscle strength indefinitely. A saturation point is reached and muscle fibres consequently atrophy, leading swiftly to clinical weakness. By assessing fasciculation amplitude serially as a surrogate of this reinnervation process, we hoped to gain insight into this process.

Fasciculation doublets have been shown to occur in biceps brachii, vastus lateralis and tibialis anterior from patients with amyotrophic lateral sclerosis (Kleine *et al.*, 2008; De Carvalho and Swash, 2013), as well as the gastrocnemius from both patients with amyotrophic lateral sclerosis and benign fasciculation syndrome (Mills, 2010; Kleine *et al.*, 2012). They are defined as the occurrence of two almost identical MU potentials, presumed to both arise from the same MU, with a very short IFI of <100 ms. The prompt succession of the second fasciculation in the doublet is thought to be due to the supernormal (i.e. superexcitable) period of the recovery cycle (Bostock *et al.*, 1995). It has been postulated that the IFI of these doublets can indicate the origin of the doublet, owing to varying characteristics of the recovery cycle at different locations along the motor neuron. Shorter IFIs (5–10 ms) are likely to arise distally in the terminal branches, whereas longer IFIs (40–80 ms) are thought to originate proximally at the soma (Kleine *et al.*, 2008). Faced with the low occurrence rate of doublets during electrical stimulation (Maathuis *et al.*, 2012), we hypothesized that the collection of vast numbers of fasciculations would be required to observe IFI peaks in these ranges. In turn, this might help to elucidate the origin of fasciculations in amyotrophic lateral sclerosis.

In this study, we set out to calibrate our novel analytical system longitudinally in the clinical context of amyotrophic lateral sclerosis disease progression. We have compared these results with control subjects who have benign fasciculation syndrome, a condition that is defined by the isolated presence of fasciculations, particularly in muscles of the lower limbs, without evidence of underlying motor neuron degeneration (Blexrud *et al.*, 1993). We aimed to identify a novel quantitative biomarker related to fasciculations that could monitor patients with amyotrophic lateral sclerosis over time. If this could be achieved, the relative simplicity of the HDSEMG technique, working alongside an automated analytical system, would make its widespread application as a clinical and research tool more viable.

## Materials and methods

### Patient recruitment

Twenty patients with amyotrophic lateral sclerosis and five patients with benign fasciculation syndrome each underwent up to seven assessments at intervals of 2 months (Table 1). Patients with amyotrophic lateral sclerosis were diagnosed with probable or definite amyotrophic lateral sclerosis using the revised El Escorial Criteria (Brooks *et al.*, 2000) within 24 months of symptom onset. Ethical approval was obtained from the East Midlands (Nottingham 1) Research Ethics Service (Ref: 17/EM/0221). Patients were recruited from the King’s College Hospital Motor Nerve Clinic between July 2017 and February 2018 and provided informed written consent before participation according to the Declaration of Helsinki. Premature withdrawal from the study in the amyotrophic lateral sclerosis group was due to either worsening physical disability (precluding travel to and from the hospital; 9/20 patients) or death (patients 2, 9 and 12). One patient with benign fasciculation syndrome withdrew early due to relocation away from London. Data from a further eight patients (six patients with amyotrophic lateral sclerosis, one with benign fasciculation syndrome and one with multifocal motor neuropathy) from our previously reported pilot study (Bashford *et al.*, 2019) were included in the cumulative plots related to IFIs.

**Table 1:** Patient characteristics

Patient number	Age (years)	Gender	Diagnosis	Site of symptom onset	Duration since symptom onset (months)	Elbow flexion power (MRC scale, 5 = normal)		Plantar flexion power (MRC scale, 5 = normal)		Number of assessments undertaken
						R	L	R	L	
1	72	M	Amyotrophic lateral sclerosis	Both ULs	34	5	5	5	5	7
2	62	M	Amyotrophic lateral sclerosis	Right LL	17	5	5	4	4	1
3	60	M	Amyotrophic lateral sclerosis	Left LL	25	5	5	5	4.3	7
4	55	M	Amyotrophic lateral sclerosis	Left LL	11	5	5	4	3	4
5	51	M	Amyotrophic lateral sclerosis	Right LL	27	5	5	5	5	7
6	68	M	Amyotrophic lateral sclerosis	Left UL	35	5	4.3	5	5	5
7	57	M	Amyotrophic lateral sclerosis	Bulbar	23	5	5	5	5	1
8	64	M	Amyotrophic lateral sclerosis	Right UL	22	1	2	5	5	4
9	66	F	Amyotrophic lateral sclerosis	Bulbar	23	5	5	5	4.3	4
10	64	M	Amyotrophic lateral sclerosis	Right UL/LL	34	3.7	5	3.7	5	7
11	66	M	Amyotrophic lateral sclerosis	Bulbar	30	5	5	5	5	7
12	61	M	Amyotrophic lateral sclerosis	Left LL	22	5	5	4.3	5	3
13	79	M	Amyotrophic lateral sclerosis	Both ULs	28	3.7	5	5	5	4
14	48	M	Amyotrophic lateral sclerosis	Bulbar	48	5	5	5	4	6
15	80	M	Amyotrophic lateral sclerosis	Right UL	21	5	5	5	5	6
16	61	M	Amyotrophic lateral sclerosis	Bulbar	13	5	5	1	5	7
17	77	F	Amyotrophic lateral sclerosis	Both LLs	24	5	5	5	5	7
18	52	M	Amyotrophic lateral sclerosis	Bulbar	31	5	5	5	5	5
19	58	M	Amyotrophic lateral sclerosis	Right UL	11	3.7	4.3	5	5	7
20	71	M	Amyotrophic lateral sclerosis	Right UL	60	3.7	5	5	5	6
21	41	M	Benign fasciculation syndrome	Right LL	27	5	5	5	5	7
22	38	M	Benign fasciculation syndrome	Left UL	24	5	5	5	5	2
23	36	M	Benign fasciculation syndrome	ULs/LLs	7	5	5	5	5	6
24	55	M	Benign fasciculation syndrome	Left UL	36	5	5	5	5	7
25	38	M	Benign fasciculation syndrome	Left LL	12	5	5	5	5	7
Summary	Amyotrophic lateral sclerosis: mean 64 years; benign fasciculation syndrome: mean 42 years	Amyotrophic lateral sclerosis: 18M/2F; benign fasciculation syndrome: 5M	20 Amyotrophic lateral sclerosis; 5 benign fasciculation syndrome	Amyotrophic lateral sclerosis: UL (37.5%), LL (32.5%), bulbar (30%); benign fasciculation syndrome: UL (50%), LL (50%)	Amyotrophic lateral sclerosis: mean 30 months; benign fasciculation syndrome 21 months	Amyotrophic lateral sclerosis: mean 4.5; benign fasciculation syndrome: mean 5	Amyotrophic lateral sclerosis: mean 4.8; benign fasciculation syndrome: mean 5	Amyotrophic lateral sclerosis: mean 4.6; benign fasciculation syndrome: mean 5	Amyotrophic lateral sclerosis: mean 4.7; benign fasciculation syndrome: mean 5	Amyotrophic lateral sclerosis: mean 5.3; benign fasciculation syndrome: mean 5.8

Muscle powers were assessed at baseline by the same clinician (J.A.B.). Scores of 4+, 4 and 4− were converted to numerical scores of 4.3, 4 and 3.7, respectively.

F: female; LL: lower limb; M: male; UL: upper limb.

### Data collection and processing

Baseline demographic data and a neurological examination were documented on the first visit. Patients were asked to report the month of onset of their symptoms (defined as the first occurrence of persistent weakness in a typical muscle group for patients with amyotrophic lateral sclerosis). As clinical measures of disease progression in patients with amyotrophic lateral sclerosis, we performed the amyotrophic lateral sclerosis-functional rating scale (Cedarbaum *et al.*, 1999), Medical Research Council (MRC) sum power score (Dyck *et al.*, 2005) and slow vital capacity (Andrews *et al.*, 2018) at each assessment. Muscle power scores of 4+, 4 and 4− were converted to numerical scores of 4.3, 4 and 3.7, respectively. All assessments took place in the Academic Neuroscience Centre, King’s College Hospital, London, UK.

At each assessment, 30-min HDSEMG recordings were taken from biceps brachii and medial gastrocnemius bilaterally. The HDSEMG data collection methods have been previously reported in detail (Bashford *et al.*, 2019). Briefly, patients were asked to relax on the examination couch with legs in a horizontal, partially flexed position and forearms prone with an elbow angle of 90–120°. Before sensor placement, the skin overlying biceps and gastrocnemius was lightly scrubbed with an abrasive gel and a 70% alcohol wipe. The sensor had 64 circular electrodes (8 × 8 grid; electrode diameter 4.5 mm; inter-electrode distance 8.5 mm), and signals were amplified by the Refa-64 EMG Recording System (TMS International BV, The Netherlands)*.* The raw HDSEMG data were stored as a proprietary Polybench file at a sampling rate of 2048 Hz per channel.

Description and validation of SPiQE’s analytical pipeline have been reported elsewhere (Bashford *et al.*, 2019). In brief, an initial screen for MU potentials was applied to each recording channel. This involved the detection of the most extreme amplitudes (positive and negative), representing the peaks and troughs of MU potentials. For each of these potentials, the channel with the greatest peak-trough amplitude difference was transferred into a ‘super-channel’. Based on manual counts, we found a linear relationship between average noise levels and the optimal amplitude inclusion threshold for fasciculation potentials. We confirmed that the optimal automated model was a noise-responsive algorithm, capable of adjusting its amplitude inclusion threshold according to the local noise level (referred to as ‘noise band’). In addition, areas of the recording with excessive noise were automatically identified and excluded from further analysis. This pipeline achieved a classification accuracy of 88% when applied to 5318 fasciculation potentials that had been identified manually. Unrelaxed muscle activity was identified by Active Voluntary IDentification, a semi-automated, flexible system built to exclude regular trains of MU potentials (Bashford *et al.*, 2020).

As a parallel neurophysiological measure, we performed the motor unit number index (MUNIX) in three muscles bilaterally (abductor pollicis brevis, biceps brachii and extensor digitorum brevis) for patients with amyotrophic lateral sclerosis and benign fasciculation syndrome (Nandedkar *et al.*, 2004; Neuwirth *et al.*, 2010; Neuwirth *et al.*, 2015). The machine used was the Natus UltraPro S100 with Synergy MUNIX software. J.A.B., the sole assessor, received formal training at a MUNIX tutorial organized by professor Weber *et al.* as a part of the European Network to Cure Amyotrophic Lateral Sclerosis conference (ENCALS, Milan, Italy) in May 2016.

### Computation and statistical analysis

Computation of fasciculation potentials was performed in MATLAB (R2014a) using specifically designed scripts. Statistical tests were performed in Prism V7.0a or R (V3.3.1). Laptops with Intel i7 (2.5 GHz) processor were used for all analyses.

Proportions were tested in R using pairwise comparison with Bonferroni correction where appropriate. Linear mixed-effect models were employed in R using the ‘lme4’ package. For all parameters, the following template formula (in R notation) for the linear mixed-effect model was used:

$$\text{lmer}(\text{depVar}\;∼1+\text{fixed}+\left(\text{fixed}|{\text{random}}_{1}/{\text{random}}_{2}\right),\;\text{data}),$$

where lmer was the linear mixed-effect regression function, depVar was the dependent variable in question (e.g. noise band or FF), fixed was a fixed effect and fixed|random_*n*_ modelled a random intercept and slope. The fixed effect was either: (i) ‘months’, the number of months elapsed into the study per patient; (ii) ‘group’, the diagnostic group (amyotrophic lateral sclerosis versus benign fasciculation syndrome; amyotrophic lateral sclerosis was the reference group; the slope part of the model was disabled); (iii) ‘muscleType’, the type of muscle (biceps versus gastrocnemius; biceps was the reference group; the slope part of the model was disabled); or (iv) ‘invPower’, an inverted MRC power score, which converted power scores 0–5 to 5–0 for the purposes of the mixed-effect model (the slope part of the model was disabled). Random effects were either: (i) ‘subject’, the patient ID (always present in the model), or (ii) ‘muscle’, the individual muscle being assessed (numerically coded; only present in the model when ‘depVar’ related to individual muscles; nested within ‘subject’).

When reporting results from the linear mixed-effect models, a fixed effect baseline (referred to as ‘baseline’) was given, which was the estimated value of the dependent variable when the fixed effect was equal to 0 (or the reference group). Inverted scores for power were reverted back to the correct scale. To calculate a *P*-value, a comparison model was created, which omitted the fixed effect in question. An analysis of variance test was performed comparing the addition of the fixed effect, which produced a *P*-value according to the chi-squared test. For multiple comparisons testing, the ‘glht’ function (generalized linear hypotheses), which is part of the ‘multcomp’ package (v1.4-10), was used in R. An additional benefit of using mixed-effect models was that missing values did not invalidate the model.

For reasons set out in the ‘Results’ section, we approached some aspects of the analysis in a different way. We assigned each muscle in the study to one of the following three groups:

- Pre-weakness: muscles that remained strong (5/5) at each study visit;
- Peri-weakness: muscles that were strong (5/5) on the first assessment and became (and remained) weak during the study; and
- Post-weakness: muscles that were weak at the start of the study.

This still allowed us to assess the chronology of changes, albeit in a less rigorous way than a continuous time scale.

### Data availability

The supporting data for this study are available from the corresponding author upon reasonable request.

## Results

### Data processing

A total of 420 (210 biceps, 210 gastrocnemius) amyotrophic lateral sclerosis and 116 (58 biceps, 58 gastrocnemius) benign fasciculation syndrome recordings were analysed. Ten biceps recordings from two patients with amyotrophic lateral sclerosis were excluded due to contamination from a Parkinsonian resting tremor (seven recordings from the right biceps of patient 3) or the electrocardiogram (three recordings from the left biceps of patient 11). The processing times for successive stages of the SPiQE pipeline are shown in Table 2. There was no difference in the proportion of recordings without voluntary activity between patients with amyotrophic lateral sclerosis and benign fasciculation syndrome (52% versus 44%, *P* = 0.17).

**Table 2:** Processing times

Stage of processing	Processing time (s): median (IQR)
1. Data extraction into MATLAB from raw data file	345 (326–367)
2. Identification of possible fasciculation potentials and creation of super-channel	946 (732–1389)
3. Confirmation of fasciculation potentials and detection of voluntary potentials	
First run	179 (139–235)
Subsequent runs (when required)	10 (8–18)

Times represent MATLAB processing for a single 30-min recording on a laptop with Intel i7 (2.5 GHz) processor.

IQR: inter-quartile range.

### Data quality

#### Noise band

There was no difference in noise band between patients with amyotrophic lateral sclerosis (2.7 μV) and benign fasciculation syndrome (2.3 μV) for both muscle types (*P* = 0.32). There was a small but significant reduction in noise band as the study progressed in patients with amyotrophic lateral sclerosis (−0.04 μV per month, *P* = 0.013) but not in patients with benign fasciculation syndrome. Biceps generated higher noise band values than gastrocnemius (3.2 versus 2.1 μV, *P* < 0.0001), consistent with a previous report of this technique (Bashford *et al.*, 2019).

#### Number of channels and total time included

There was no difference in the number of channels included between patients with amyotrophic lateral sclerosis (19.1/36) and benign fasciculation syndrome (18.7/36) for both muscle types (*P* = 0.53). There was also no difference in the total time included between patients with amyotrophic lateral sclerosis (26.4 min) and benign fasciculation syndrome (27.2 min) for both muscle types (*P* = 0.59).

### Established clinical and neurophysiological parameters

#### Amyotrophic lateral sclerosis-functional rating scale

**Figure 1:**
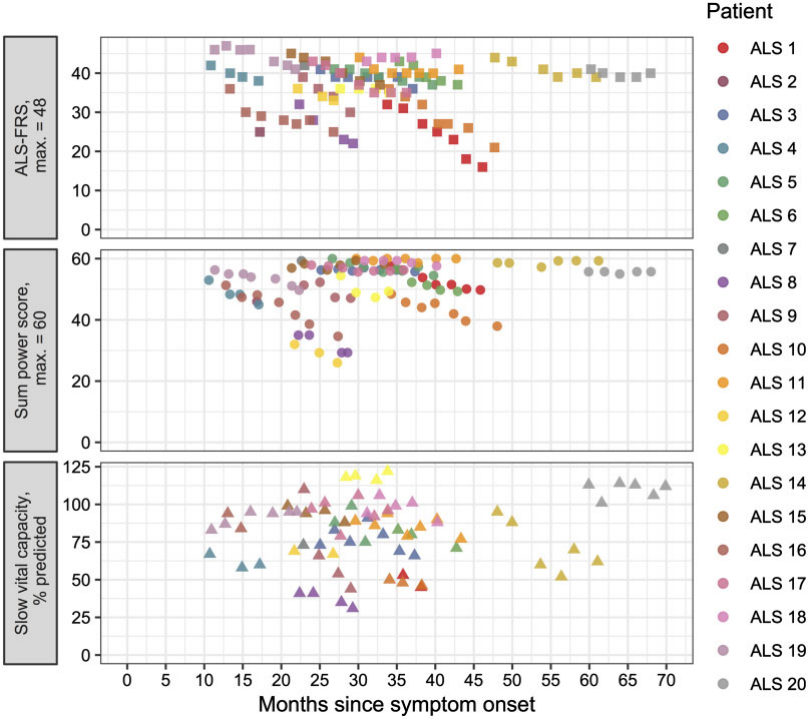
**Change in clinical parameters over time.** Plots of amyotrophic lateral sclerosis-functional rating scale (*top*, square symbols), sum MRC power score (*middle*, circle symbols) and slow vital capacity (*bottom*, triangle symbols) per patient (colour coded as per legend) against months elapsed since symptom onset (see ‘Results’ section for statistical analyses).

Only patients with amyotrophic lateral sclerosis had the amyotrophic lateral sclerosis-functional rating scale performed at each visit (Fig. 1). The baseline was 39.1/48 with a strongly significant decrease of 0.65 points per month (*P* < 0.0001). This indicated that the patients enrolled in this study had a 35% slower progression than average, which is typically reported as a fall of 1 point on the amyotrophic lateral sclerosis-functional rating scale per month (Thakore *et al.*, 2018).

#### Muscle power score

Only patients with amyotrophic lateral sclerosis had a sum MRC power score recorded at each visit (Fig. 1). The baseline was 53.1/60 with a strongly significant decrease of 0.5 points per month (*P* < 0.0001). There was no difference in muscle power between biceps and gastrocnemius in patients with amyotrophic lateral sclerosis (baseline = 4.45/5 for both muscle types, *P* = 0.93).

#### Slow vital capacity

Only patients with amyotrophic lateral sclerosis had a slow vital capacity recorded at each visit (Fig. 1). Four out of 20 patients either declined slow vital capacity testing or had significant facial weakness making the results unreliable. Two out of 20 patients developed the need for non-invasive ventilation during the course of the study, and slow vital capacity testing was not continued. The baseline was 84.0% of predicted with a strongly significant decrease of 1.7% per month (*P* < 0.0001).

#### Motor unit number index

Baseline MUNIX-abductor pollicis brevis was lower in patients with amyotrophic lateral sclerosis compared to patients with benign fasciculation syndrome (101 versus 192, *P* = 0.0007) with a decline of –3.4% per month in the amyotrophic lateral sclerosis cohort (*P* < 0.0001). Similarly, baseline MUNIX-biceps was lower in patients with amyotrophic lateral sclerosis compared to patients with benign fasciculation syndrome (168 versus 279, *P* < 0.0001) with a decline of –3.0% per month in the amyotrophic lateral sclerosis cohort (*P* < 0.0001). These rates of decline in patients with amyotrophic lateral sclerosis were comparable to a previous longitudinal study for these upper limb muscles (Neuwirth *et al.*, 2015). Moreover, among patients with amyotrophic lateral sclerosis, there was a significant decline in MUNIX-biceps in both strong muscles [–2.6% of baseline (216) per month, *P* < 0.0001] and weak muscles [–6.2% of baseline (125) per month, *P* = 0.0005], consistent with previous reports that MUNIX tracks pre-symptomatic decline (Fukada *et al.*, 2016; Neuwirth *et al.*, 2017). Baseline MUNIX-extensor digitorum brevis was lower in patients with amyotrophic lateral sclerosis compared to patients with benign fasciculation syndrome (41.7 versus 100.8, *P* = 0.0048) with a decline of –2.6% per month in the amyotrophic lateral sclerosis cohort (*P* = 0.0268). Overall, MUNIX was reliable in all three muscles as a monitoring tool in patients with amyotrophic lateral sclerosis.

### Experimental fasciculation parameters

#### Fasciculation frequency

**Figure 2:**
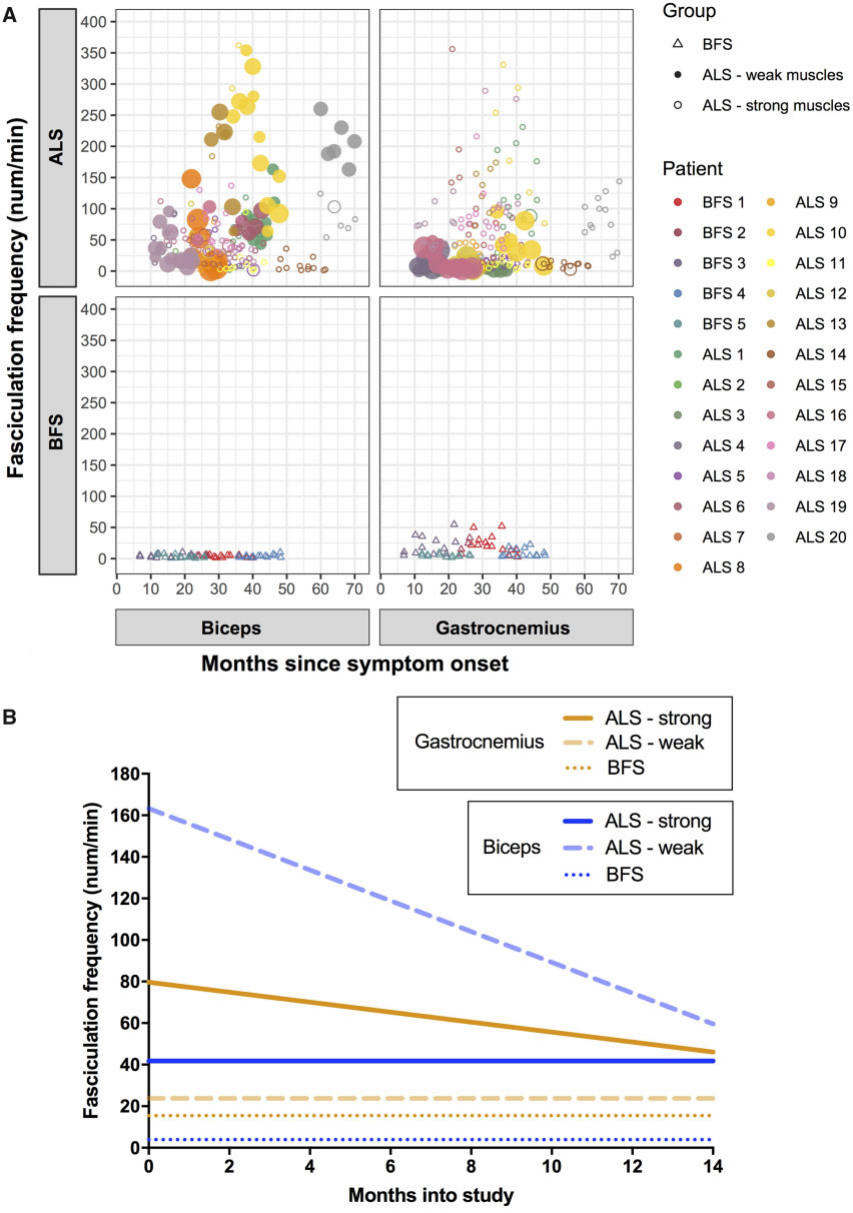
**Change in FF over time.** (**A**) FF against months since symptom onset per muscle per patient group. Individual patients are colour coded (see legend). Weak amyotrophic lateral sclerosis muscles are filled circles, and strong amyotrophic lateral sclerosis muscles are clear circles. The larger the symbol, the weaker the muscle at the time of recording. (**B**) Representation of FF over time for each muscle group, based on results from linear mixed-effect models (see ‘Results’ section).

We analysed biceps and gastrocnemius separately while obtaining comparisons between the two disease groups (Fig. 2A). For patients with amyotrophic lateral sclerosis, the FF baselines were 62.6/min (biceps) and 51.8/min (gastrocnemius) with the significant declines of –2.3/min per month (biceps; *P* < 0.0001) and –1.8/min per month (gastrocnemius; *P* < 0.0001). For patients with benign fasciculation syndrome, the baselines were 4.0/min (biceps) and 15.5/min (gastrocnemius) with no change over time (biceps, *P* = 0.286; gastrocnemius, *P* = 0.306). The differences in baselines between the two patient groups were significant for biceps (*P* = 0.031) but not for gastrocnemius (*P* = 0.088).

Subsequently, we focused on the amyotrophic lateral sclerosis group. Having shown that both FF and sum muscle power scores decreased linearly over time, we expected a direct linear correlation between FF and individual muscle power scores. This was the case for gastrocnemius (FF = 58.7/min in muscles with the power of 5/5, with a decrease of 12.2/min for each unit drop in MRC power scale, *P* = 0.02) but not for biceps (*P* = 0.19). This led us to suspect that a non-linear relationship might better describe the relationship between these two parameters in biceps. Interestingly, when the strong biceps muscles were removed from the model, there was a significant FF decline of –41.6/min for each unit drop in the MRC power scale (*P* = 0.0032).

To examine this further, we tested whether the presence of muscle weakness in patients with amyotrophic lateral sclerosis influenced the change in FF over time. For strong (5/5) biceps muscles in patients with amyotrophic lateral sclerosis (124 observations from 26 muscles in 15 patients), the baseline FF was 41.8/min with no significant change over time (−0.8/min per month, *P* = 0.60). For weak (4+/5 or weaker) biceps muscles in patients with amyotrophic lateral sclerosis (44 observations from 8 muscles in 6 patients), the baseline FF was 163.3/min with a significant decrease of 7.6/min per month (*P* = 0.003). For strong gastrocnemius muscles in patients with amyotrophic lateral sclerosis (162 observations from 29 muscles in 16 patients), the baseline FF was 79.7/min with a significant decrease of 2.4/min per month (*P* < 0.0001). For weak gastrocnemius muscles in patients with amyotrophic lateral sclerosis (31 observations from 8 muscles in 6 patients), the baseline FF was 23.8/min with a non-significant decrease of 1.4/min per month (*P* = 0.11). These results are summarized in Fig. 2B.

Although benign fasciculation syndrome is not considered a prodrome of amyotrophic lateral sclerosis, our interpretation of these results relied on the assumption that the baseline FF in patients with benign fasciculation syndrome represented the amyotrophic lateral sclerosis baseline *prior to disease onset*. For biceps, FF in strong amyotrophic lateral sclerosis muscles was 10× greater than the benign fasciculation syndrome baseline, while FF in weak muscles started at levels 40× greater than the benign fasciculation syndrome baseline. Over the 14 months of the study, FF decreased in weak muscles at a rate three times faster than average. This supported our suspicion that biceps FF was non-linear, first rising steadily from a pre-morbid baseline in strong muscles and subsequently falling as weakness ensued. Given that there was no significant change in biceps FF over the 14 months of the study in strong amyotrophic lateral sclerosis muscles, we hypothesize that the rising phase is slow, perhaps starting many years before clinical weakness. In contrast to biceps, gastrocnemius demonstrated a significant decline in FF in strong muscles, which plateaued in weak muscles.

#### Fasciculation amplitude

**Figure 3:**
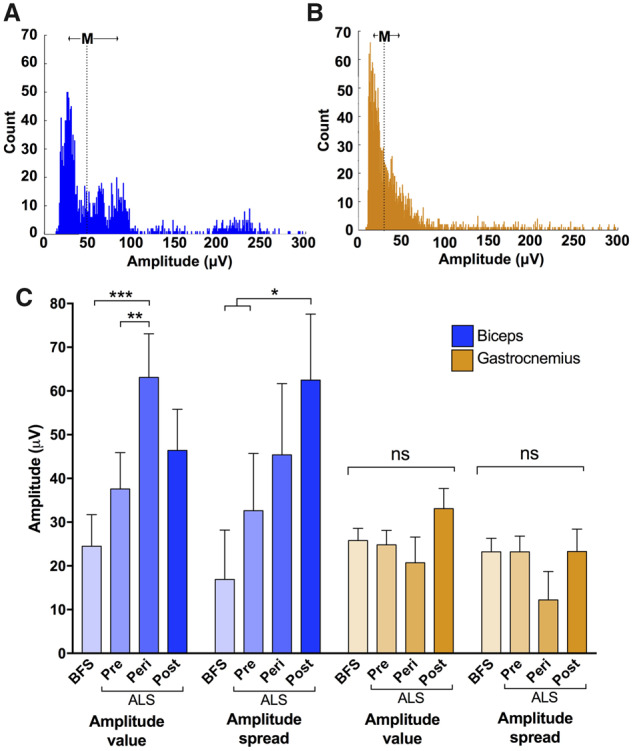
**Comparison of amplitude parameters.** Representative amplitude histograms from individual 30-min recordings from (**A**) biceps of a patient with amyotrophic lateral sclerosis and (**B**) gastrocnemius of a patient with benign fasciculation syndrome. Note multiple peaks in ‘a’, typical of an amyotrophic lateral sclerosis recording. ‘M’ depicts median amplitude and arrows indicate inter-quartile range (IQR). Bin width = 1 μV. (**C**) The average amplitude value (median) and amplitude spread (IQR) have been calculated for each 30-min recording. Statistical comparisons were made using linear mixed-effect models with group (benign fasciculation syndrome/pre/peri/post) as fixed effect. ‘Pre’ refers to pre-weakness (i.e. amyotrophic lateral sclerosis muscles that remained strong throughout the whole study), ‘peri’ refers to peri-weakness (i.e. amyotrophic lateral sclerosis muscles that transitioned from strong to weak during the course of the study) and ‘post’ refers to post-weakness (i.e. amyotrophic lateral sclerosis muscles that began the study weak). Boxes and whiskers display means and standard error. *Post hoc* multiple comparison testing was performed using Holm-Bonferroni correction in R.

Fasciculation amplitude was employed as an easily quantifiable surrogate of the reinnervation process. When we observed the amplitude histograms of individual 30-min recordings, we noticed a particular pattern of multiple high-amplitude peaks in patients with amyotrophic lateral sclerosis but never in patients with benign fasciculation syndrome (Fig. 3A and B). We believe that these peaks represent the increased fasciculation rate of a subset of MUs rendered hyperexcitable by the disease process. We extracted the average amplitude value (median) and a measure of amplitude spread (inter-quartile range) from each recording to quantify this distinguishing feature. For biceps, the amplitude was higher in patients with amyotrophic lateral sclerosis (42.7 μV) than in patients with benign fasciculation syndrome (24.5 μV, *P* = 0.042). However, for gastrocnemius, there was no difference between the two groups (25.9 versus 25.8 μV, *P* = 0.99). For amplitude spread, there was a trend for higher values in amyotrophic lateral sclerosis (40.4 μV) compared to benign fasciculation syndrome (23.4 μV), although this failed to reach significance (*P* = 0.081). Similarly for gastrocnemius, there was no difference in amplitude spread between the two groups (22.4 versus 21.4 μV, *P* = 0.80).

Next, we assessed whether fasciculation amplitude changed over time in patients with amyotrophic lateral sclerosis. For biceps, the baseline was 37.1 μV for strong muscles and 52.5 μV for weak muscles, neither showing a significant change over time. For gastrocnemius, the baseline was 25.7 μV for strong muscles and 31.3 μV for weak muscles, again showing no significant change over time. The rise in baseline amplitudes between strong and weak muscles, particularly in biceps, warranted further exploration. We therefore compared the baseline amplitudes for three categories of muscles: pre-, peri- and post-weakness (see ‘Materials and methods’ for explanation). There was a significant increase in median amplitude in the biceps peri-weakness group compared to the pre-weakness amyotrophic lateral sclerosis group and the benign fasciculation syndrome group (*P* < 0.01; Fig. 3C), consistent with MUs that had undergone reinnervation. In addition, there was increased amplitude spread in the biceps post-weakness group compared to the pre-weakness amyotrophic lateral sclerosis group and the benign fasciculation syndrome group (*P* < 0.05). There were no differences between these three weakness groups and the benign fasciculation syndrome group in the gastrocnemius recordings. This suggested that the fasciculating MUs from the biceps of patients with amyotrophic lateral sclerosis had undergone varying degrees of reinnervation dependent on the stage of disease, whereas those from gastrocnemius had not.

#### Inter-fasciculation intervals

**Figure 4:**
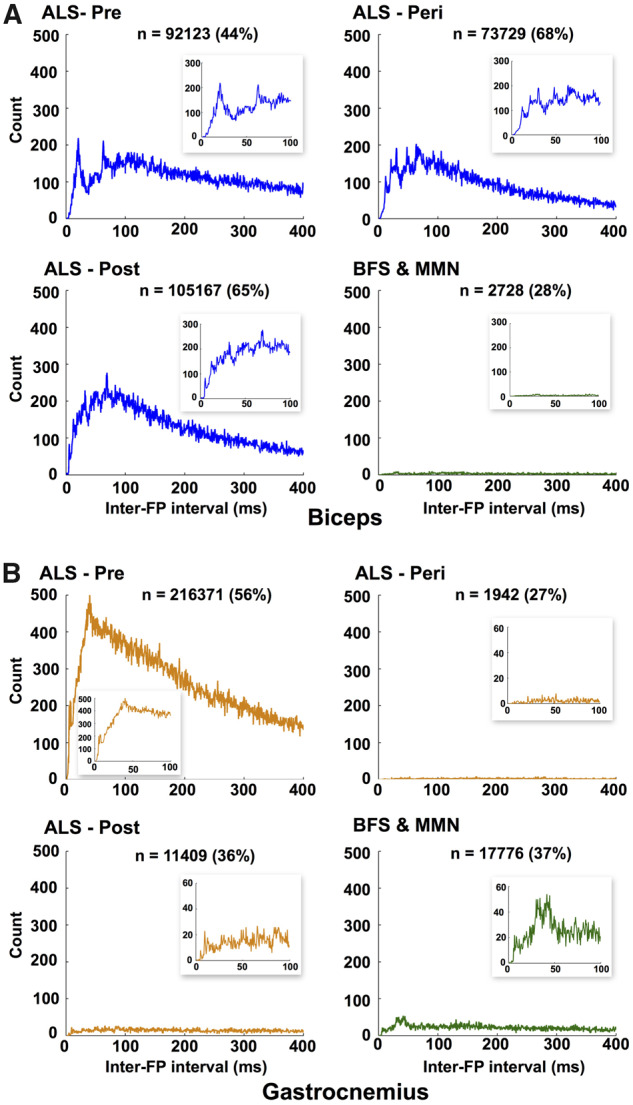
**Analysis of IFIs.** Histogram of intervals between successive FPs in the ‘super-channel’ (see ‘Materials and methods’ section for explanation) for (**A**) biceps and (**B**) gastrocnemius. These represent intervals between FPs arising from different MUs. FPs have been recorded from bilateral muscles of 26 patients with amyotrophic lateral sclerosis (1–7 assessments per patient), 6 patients with benign fasciculation syndrome (2–7 assessments per patient) and 1 patient with multifocal motor neuropathy (6 assessments). Bin width = 0.5 ms. *x*-Axis cut-off at 400 ms. *n* = number of IFIs detected in range 0–400 ms (percentage of total number within each group given in brackets). Insets show zoomed results between 0 and 100 ms. Note varying *y*-axis scales of insets. FP: fasciculation potential.

By assessing the IFIs between successive fasciculation potentials from pre-, peri- and post-weakness amyotrophic lateral sclerosis muscles, we have shown that in biceps (466 346 IFIs in total) there were multiple peaks in the sub-100 ms range, whereas in gastrocnemius (412 501 IFIs in total) only a small peak ∼10 ms was present in strong muscles (Fig. 4). Moreover, the most discrete peaks at 20 and 70 ms were seen in strong amyotrophic lateral sclerosis biceps muscles, highlighting the importance of this finding in pre-symptomatic disease. In patients with benign fasciculation syndrome, fewer fasciculations (9633 for biceps and 46 971 for gastrocnemius) were present. Despite this, there was a peak at ∼40 ms from the gastrocnemius recordings of patients with benign fasciculation syndrome, consistent with findings from previous studies using needle EMG (Mills, 2010) and HDSEMG (Kleine *et al.*, 2008). Although the sheer number of analysed fasciculation potentials precluded confirmatory morphological analysis, these peaks are consistent with fasciculation doublets.

**Figure 5:**
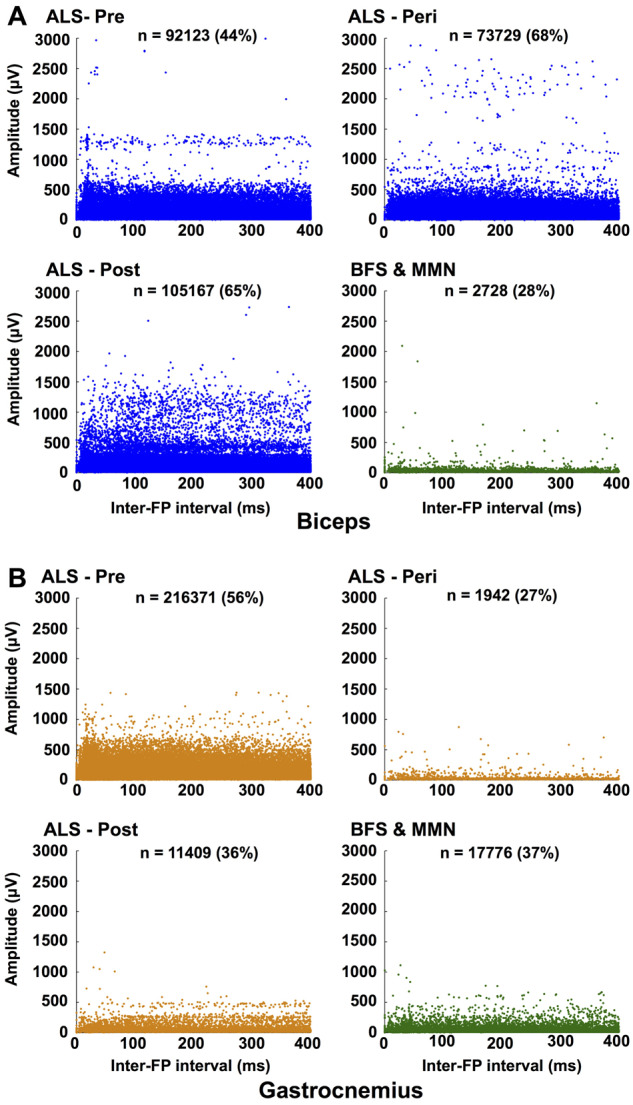
**Relationship between IFIs and FP amplitude.** Scatterplots for ‘amyotrophic lateral sclerosis-pre-weakness’, ‘amyotrophic lateral sclerosis-peri-weakness’, ‘amyotrophic lateral sclerosis-post-weakness’ and ‘benign fasciculation syndrome and multifocal motor neuropathy’ for (**A**) biceps and (**B**) gastrocnemius (see ‘Materials and methods’ section for the explanation of groups). For each interval between two successive fasciculations, each FP amplitude is plotted separately. *x*-Axis cut-off at 400 ms. *n* = number of intervals detected in range 0–400 ms (percentage of total number within each group given in brackets). FP: fasciculation potential.

For each amyotrophic lateral sclerosis weakness group (pre/peri/post) and the control group (benign fasciculation syndrome and multifocal motor neuropathy), we compared all IFIs in the range 0–400 ms against the fasciculation potential amplitudes within each pair (Fig. 5). In biceps, the ‘amyotrophic lateral sclerosis-pre’ group demonstrated a band of increased amplitudes ∼1300 μV, likely to represent the earliest reinnervating MUs. Around the time that clinical weakness was developing (‘amyotrophic lateral sclerosis-peri’ group) in biceps muscles, fasciculation amplitudes >2000 μV became frequent, suggesting that the fasciculating MUs had undergone more reinnervation than at an earlier stage of disease. The absence of these high-amplitude fasciculation potentials in the ‘amyotrophic lateral sclerosis-post’ group suggests that the death of the most reinnervated MUs played a significant part in the development of clinical weakness. This is unsurprising given that reinnervation is a compensatory mechanism designed to maintain muscle strength for as long as possible. Finally, the ‘amyotrophic lateral sclerosis-post’ group had considerably more intermediate amplitude fasciculations (500–1500 μV) than earlier stages of disease. This indicated that a greater number of MUs had had time to undergo reinnervation, but due to the overwhelming loss of MU numbers at this stage of disease, this was not enough to maintain muscle power.

For gastrocnemius, interpretation was hampered by the relative lack of IFIs in the ‘amyotrophic lateral sclerosis-peri’, ‘amyotrophic lateral sclerosis-post’ and ‘benign fasciculation syndrome and multifocal motor neuropathy’ groups. Despite this, the absence of high-amplitude fasciculation potentials suggests one of three possible conclusions. It could be that either gastrocnemius MUs do not undergo the same degree of reinnervation as in biceps; its MUs undergo reinnervation at a very early stage of disease and those reinnervating MUs die early; or the reinnervating MUs do not fasciculate.

## Discussion

The most striking implication from these results was the rise and subsequent fall of FF in amyotrophic lateral sclerosis biceps muscles. This non-linear pattern had been previously suggested after statistically modelling fasciculation counts using muscle ultrasound (Vazquez-Costa *et al.*, 2018) and might explain why a previous surface EMG study of FF did not show a significant linear change over time (De Carvalho and Swash, 2016). To help establish this pattern, we divided the data into strong and weak muscles, a clinically meaningful and unambiguous distinction. Furthermore, due to the serial nature of the data, we were able to divide each muscle into pre-weakness, peri-weakness and post-weakness groups. This allowed us to assess the chronology of disease by equating these groups to early, middle and late stages of disease, respectively. This was only possible due to the anatomical specificity of the HDSEMG technique, which is a major strength in this setting.

In this study, the highest fasciculation frequencies were found in the amyotrophic lateral sclerosis biceps muscles that had recently become weak. In amyotrophic lateral sclerosis, we can reasonably hypothesize that the two main contributing factors to FF are the size of the *affected* MU pool and the relative degree of hyperexcitability. As others have shown previously (Neuwirth *et al.*, 2017), MUNIX confirmed the declining size of the *viable* MU pool over time in biceps muscles, even while muscles remained strong (albeit at a slower rate than weak muscles). However, it remains unknown what proportion of MUs are affected (and therefore hyperexcitable) at a given stage of the disease. An appealing assumption is that this proportion is relatively constant over time, consistent with an equilibrium between the rate that healthy MUs become affected and the death rate of already affected ones. In this scenario, we postulate that it was principally an increase in the relative hyperexcitability of affected MUs driving the postulated rising phase in FF in strong amyotrophic lateral sclerosis muscles. This is consistent with results from the excitability testing of human motor axons, showing an increased strength–duration time constant (as a measure of increased Na+ conductance) and an altered threshold electrotonus (as a measure of impaired K+ conductance) in amyotrophic lateral sclerosis (Bostock *et al.*, 1995; Mogyoros *et al.*, 1998; Burke *et al.*, 2001; Kanai *et al.*, 2006; Iwai *et al.*, 2016). Importantly, improvement in these excitability measures with riluzole and retigabine has emphasized their pathological significance (Vucic *et al.*, 2013a; Kovalchuk *et al.*, 2018).

**Figure 6:**
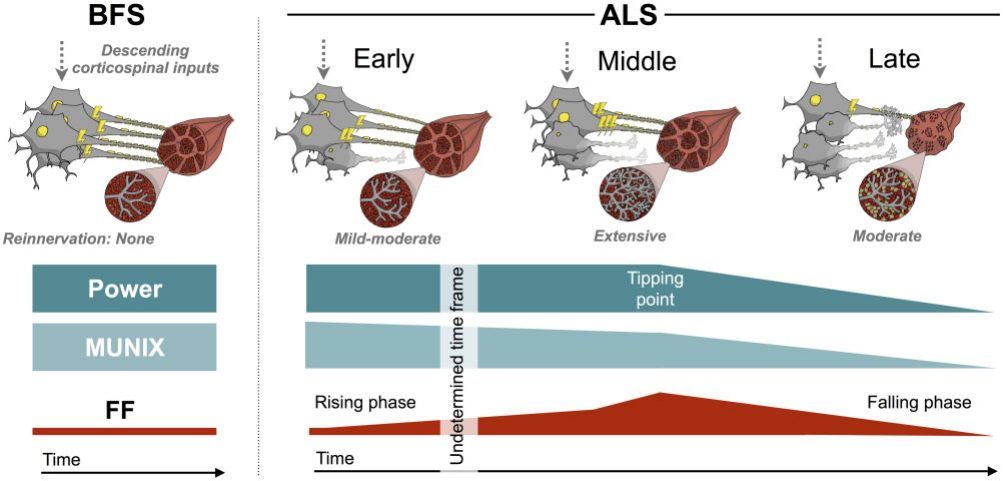
**Proposed model—the rise and fall of fasciculations in individual amyotrophic lateral sclerosis muscles.** This is our proposed model of the interactions between muscle power, size of viable MU pool (as assessed by MUNIX) and FF in benign fasciculation syndrome and three stages of disease in amyotrophic lateral sclerosis. The diagrams depict the dynamic changes in MU architecture and relative hyperexcitability (depicted by electric bolts) as a consequence of motor neuron degeneration and MU loss. In benign fasciculation syndrome, there is global hyperexcitability affecting all MUs to a similar degree in the absence of motor neuron degeneration. In early amyotrophic lateral sclerosis, a subset of MUs are hyperexcitable, MU loss has begun and mild–moderate compensatory reinnervation has occurred. Due to the stability of biceps FF in strong muscles over 14 months (at a firing rate ∼10× greater than the benign fasciculation syndrome baseline), the rising phase is hypothesized to begin many years before muscle weakness first appears. It is postulated that towards the latter end of the rising phase, the rate of increase in FF speeds up, so that by the onset of weakness, FF is ∼40× the benign fasciculation syndrome baseline. In the middle stage, the ongoing loss of MUs has promoted extensive reinnervation of surviving MUs, which then become hyperexcitable themselves. This compensatory mechanism leads to fasciculations of greater amplitude and allows muscles to remain strong by staving off muscular atrophy. However, a tipping point is reached, whereby these compensatory mechanisms saturate, leading to the onset of muscle atrophy and weakness. In late amyotrophic lateral sclerosis, the death of the most reinnervated MUs leads to worsening muscle atrophy and weakness. The relentless loss of MUs drives the falling FF. Evidence of doublets with IFIs in the 20–80 ms range is consistent with the supernormal period of MU subtypes (fast-slow), supporting a proximal origin of fasciculations at the soma. Consequently, throughout all stages of amyotrophic lateral sclerosis and in benign fasciculation syndrome, the degree of hyperexcitability of the lower motor neuron is likely to be driven and/or influenced by descending corticospinal inputs.

The subsequent decline in FF can be attributed to the relentlessly shrinking MU pool. There is clear evidence of this in Fig. 5A, which shows the death of very high-amplitude MUs (>2000 μV) as muscles weaken. With fewer MUs present and a relative hyperexcitability that presumably saturates [or even reverses, as suggested in a recent study of SOD1^G93A^ mice (Martínez-Silva *et al.*, 2018)], the natural conclusion is an attrition in fasciculation firing. This theory is summarized in Fig. 6.

We observed several key differences between the behaviour of gastrocnemius and biceps in patients with amyotrophic lateral sclerosis. Gastrocnemius demonstrated a linear decline in FF among strong muscles, which levelled off in weak muscles. In addition, we observed no significant change in fasciculation amplitude median or inter-quartile range in gastrocnemius, providing no evidence for significant reinnervation of fasciculating MUs. Also, in contrast to biceps, we saw very few IFIs in the sub-100 ms range, indicating that MUs supplying gastrocnemius were generally less hyperexcitable.

In an attempt to explain these different patterns of fasciculation firing, it is important to consider some physiological differences between biceps and the plantar flexors (gastrocnemius and soleus). Burke suggested that the ratio of fast-twitch:slow-twitch MU subtypes in cat gastrocnemius was high (∼3.5:1) (Burke, 1967; Burke *et al.*, 1970), while others have found no slow MUs in gastrocnemius (Devanandan *et al.*, 1965). In human biceps, the proportion of fast-twitch:slow-twitch MUs is thought to be closer to 1:1 based on the even distribution of muscle fibre subtypes (Srinivasan *et al.*, 2007). Notably, it is recognized in mouse models of amyotrophic lateral sclerosis that fast-twitch MUs are the most susceptible to disease, owing in part to their higher energy requirements, while slow-twitch MUs are the principle reinnervating units (Frey *et al.*, 2000; Pun *et al.*, 2006). Soleus, a plantar flexor deep to gastrocnemius, has a much greater proportion of slow-twitch muscle fibres (70–80%) (Gollnick *et al.*, 1974), suggesting that it may be relatively spared in amyotrophic lateral sclerosis, able to maintain plantar flexion strength for much longer than gastrocnemius could do alone. Therefore, we hypothesize that the predominance and subsequent loss of susceptible fast-twitch MUs in gastrocnemius led to the falling FF pattern observed in this study. The relative lack of reinnervating slow-twitch MUs in gastrocnemius might also explain the stable fasciculation amplitude profile indistinguishable from benign fasciculation syndrome muscles.

The multiple high-amplitude peaks seen in Fig. 3A, characteristic of amyotrophic lateral sclerosis recordings, indicate that fasciculations might arise from a subset of hyperexcitable MUs that have undergone reinnervation. The absence of this pattern in benign fasciculation syndrome suggests a global hyperexcitability affecting all MUs in this benign disorder. However, it is important to consider other key influences on fasciculation amplitude, including the depth of the MU, the effects of muscle atrophy and the size of the MU (Barkhaus and Nandedkar, 1994). Future studies should attempt to elucidate the relative contributions of these factors by correlating surface EMG and appropriate imaging techniques, such as ultrasound and magnetic resonance imaging (Vazquez-Costa *et al.*, 2018; Whittaker *et al.*, 2019).

The analysis of IFIs had significant strength in numbers (>900 000 fasciculations analysed); however, due to prohibitive computational expense, this was offset by a lack of precision regarding the morphological characteristics of each fasciculation. We concluded that any peak in IFIs that was not consistent with random firing (in this case a Poisson distribution) could indicate the presence of fasciculation doublets. Previous studies have suggested that fasciculation doublets with an interval of 5–10 ms represent a distal origin in the terminal branches of the MU, whereas intervals in the 60–80 ms range represent origin at the motor neuron soma (Kleine *et al.*, 2008). We did not observe many intervals in the 5–10 ms range; however, in biceps, we observed several peaks in the 20–80 ms range.

To help explain this finding, it is important to emphasize that MUs are divided into subtypes, including fast-fatigable, fast-intermediate and slow-twitch units (Pun *et al.*, 2006). It is pertinent that these subtypes demonstrate temporal variations in their recovery cycles, which in turn dictate their specialist firing characteristics (Burke, 1967). An important determinant of the maximum firing frequency of lower motor neurons at the soma is the duration of the afterhyperpolarization phase of the recovery cycle, estimated to cover a broad range between 20 and 150 ms (Eccles *et al.*, 1958; Burke *et al.*, 2001). The hyperexcitable supernormal period arises from intermodal capacitance and is thought to precede the afterhyperpolarization (Stys and Waxman, 1994). Therefore, it is reasonable to suspect that a motorneuron with a supernormal period in the 20–40 ms range might occasionally produce fasciculation pairs with an IFI of 20–40 ms simply by chance. Our data are consistent with a spectrum of MU subtypes, ranging from the slowest (IFI peak 60–80 ms) to fastest (IFI peak 20–40 ms). This interpretation is consistent with a proximal origin of fasciculations at the soma, possibly driven or modified by upstream corticospinal inputs (Mills and Nithi, 1997; Vucic *et al.*, 2013b). Moreover, in strong amyotrophic lateral sclerosis biceps muscles, the fastest MUs produced a higher number of amplitudes >1000 μV (Fig. 5A), suggesting the co-occurrence of reinnervation and doublet formation in a subset of MUs. Importantly, the presence of these IFI peaks in the earliest stages of disease highlights their potential predictive or diagnostic utility.

A major limitation of this study was the 90% male predominance among the amyotrophic lateral sclerosis cohort, limiting its generalizability among female patients. There was a higher than expected withdrawal rate of 45%, leading to 25% fewer assessments than planned, consistent with reported withdrawal rates in other longitudinal studies of patients with amyotrophic lateral sclerosis (Neuwirth *et al.*, 2015, De Carvalho and Swash, 2016). The progressive immobility associated with amyotrophic lateral sclerosis means that any longitudinal study that relies on burdensome hospital assessments will always be biased towards patients with less aggressive disease. This study was no exception with a 35% slower rate of progression than average. Arguably, the perfect biomarker would be one that could be acquired by patients and their carers in their own homes, allowing for data acquisition on a regular basis (e.g. weekly), while maintaining its sensitivity at tracking the underlying disease process. In that way, the assessment of strong muscles in individuals with an established amyotrophic lateral sclerosis diagnosis might prove to be an effective way of observing the pre-manifest phase (prior to symptoms or signs of disease) or prodromal phase (after possible symptoms or signs of disease) within individual muscles (Benatar *et al.*, 2019). This approach can complement the monitoring of pre-symptomatic ‘at-risk’ individuals, known to harbour pathogenic mutations, and waiting for their phenoconversion (Benatar and Wuu, 2012).

## Conclusion

This longitudinal study of patients with amyotrophic lateral sclerosis and benign fasciculation syndrome over 14 months provides the most comprehensive quantification of fasciculations in biceps and gastrocnemius to date. By applying an automated analytical method (SPiQE) to over 250 hours of serial HDSEMG recordings, we have shown that FF, fasciculation amplitude and IFIs can aid our understanding of motor neuron health in amyotrophic lateral sclerosis. From this, we have proposed a model of how muscle power, the size of the MU pool and FF interact over the course of disease. Of particular note, the postulated rise and fall of FF over time demonstrated the most promise as a useful disease-monitoring biomarker, warranting external validation and further clinical calibration.

## Abbreviations

FF: fasciculation frequency
HDSEMG: high-density surface electromyography
IFI: inter-fasciculation interval
MRC: medical research council
MU: motor unit
MUNIX: motor unit number index
SPiQE: Surface Potential Quantification Engine
